# MR Image Based Approach for Metal Artifact Reduction in X-Ray CT

**DOI:** 10.1155/2013/524243

**Published:** 2013-11-04

**Authors:** Andras Anderla, Dubravko Culibrk, Gaspar Delso, Milan Mirkovic

**Affiliations:** ^1^Department of Industrial Engineering and Management, Faculty of Technical Sciences, University of Novi Sad, Trg Dositeja Obradovica 7, 21000 Novi Sad, Serbia; ^2^Department of Medical Imaging, University Hospital, Rämistrasse 100, 8091 Zürich, Switzerland

## Abstract

For decades, computed tomography (CT) images have been widely used to discover valuable anatomical information. Metallic implants such as dental fillings cause severe streaking artifacts which significantly degrade the quality of CT images. In this paper, we propose a new method for metal-artifact reduction using complementary magnetic resonance (MR) images. The method exploits the possibilities which arise from the use of emergent trimodality systems. The proposed algorithm corrects reconstructed CT images. The projected data which is affected by dental fillings is detected and the missing projections are replaced with data obtained from a corresponding MR image. A simulation study was conducted in order to compare the reconstructed images with images reconstructed through linear interpolation, which is a common metal-artifact reduction technique. The results show that the proposed method is successful in reducing severe metal artifacts without introducing significant amount of secondary artifacts.

## 1. Introduction

Trimodality systems are capable of acquiring computed tomography (CT), positron emission tomography (PET), and magnetic resonance (MR) datasets in a single session. This offers new possibilities to use the advantages of all three scanners. One of them is the reduction of metal artifacts in CT images using complementary MR images.

Computed tomography (CT) imaging systems create cross-sectional images of soft tissue, internal organs, bones, and blood vessels. Although these systems provide detailed information about patient's internal structure, they do not provide information on tissue function which is important for differentiation between normal and pathologic functions [1].

Positron emission tomography (PET) provides information about functional processes within human body. However, PET images are not enough for precise localization of organs or lesions. Therefore, superimposed CT and PET images can lead to accurate diagnosis and more precise information [2]. Another benefit from combining these two modalities is that CT images can be used to generate attenuation correction factors in PET emission data [3].

Magnetic resonance (MR) imaging creates images of atoms' nuclei using the property of nuclear magnetic resonance. This allows MRI systems to extract more detailed information about the human body than is possible to get with X-rays. Artifacts occur in MR images in the presence of ferromagnetic metal, but the study conducted by Eggers et al. [4] shows that dental fillings did not reduce the quality of images from an MRI sequence. Certain attempts were made in order to use MR images for the creation of attenuation maps for PET images, such as the method presented by Martinez-Moller et al. [5]. 

Metallic implants in CT images cause dark and bright streaking artifacts because of the high atomic number of metal. Low-energy X-ray photons passing through these objects are highly attenuated, and this leads to loss of projection data.

In addition to this type of effects, different physical phenomena are reported in the literature, which lead to CT images affected by the artifacts. Some of them are beam hardening, noise, Compton scattering, partial volume effect, cardiac, and respiratory motion [1, 2]. Beam hardening artifacts occur since the image reconstruction algorithm does not take into account the polychromatic nature of X-ray beam. Low energy photons in an X-ray beam are preferentially absorbed and as a result, the beam's energy gradually increases. When the beam is harder, it is less attenuated further. Yan et al. [6] presented a reconstruction algorithm using a polychromatic X-ray beam and they were able to remove a substantial portion of beam hardening artifacts.

There are several types of image noise that can affect the imaging process, but the ultimate source is the random, statistical noise [7]. The author showed that this type of noise can be avoided by increasing the X-ray exposure.

Compton scattering is a phenomenon, which arises from the fact that when X-ray photons pass through a matter, they are deviated from their original direction. These photons are then useless for the reconstruction. Artifacts in the direction of highest attenuation are the strongest artifacts [8].

Partial volume effect is present when a voxel is partially filled with certain substance. When the voxel is reconstructed, it represents the weighted average of the attenuations for all substances in that particular voxel. Decreasing slice thickness can decrease the partial volume effect. Several algorithms have been proposed for the correction of these artifacts [9]. Although these methods gave promising results in computer simulations, they failed when applied to clinical data.

Motion during the scanning process causes inconsistencies in the measurements. There are several types of motion that can occur in such conditions: discrete motion, pulsating motion, and continuous motion. All of these cause different types of artifacts. If the acquisition speed is increased, the probability of motion artifacts is decreased. An algorithm which tries to solve this issue is presented by Glover and Pelc [10].

The presence of metal objects leads to the amplification of the effects of listed phenomena. Metal objects are removed from the patient, if possible, but usually this is not the case in modern medical practice. Different methods have been proposed for metal artifact reduction in the literature. The easiest solution for metal artifact avoidance would be to use less attenuating materials, such as titanium. Another possibility is to use X-ray beams with higher energy, but this approach does not give very good results and also the patient is exposed to a larger dose of radiation. Therefore, numerous techniques have been proposed aimed at the elimination or reduction of the effects caused by metallic objects. These approaches can be divided into two groups: implicit and explicit methods. Implicit methods try to suppress artifacts without the need to apply algorithmic mathematical methods for metal-artifact correction. Their drawback is that they have limited applicability. On the other hand, explicit methods are more general and they represent the main focus of researchers. An extensive list of existing techniques and methods can be found in [11].

**Figure 1:**
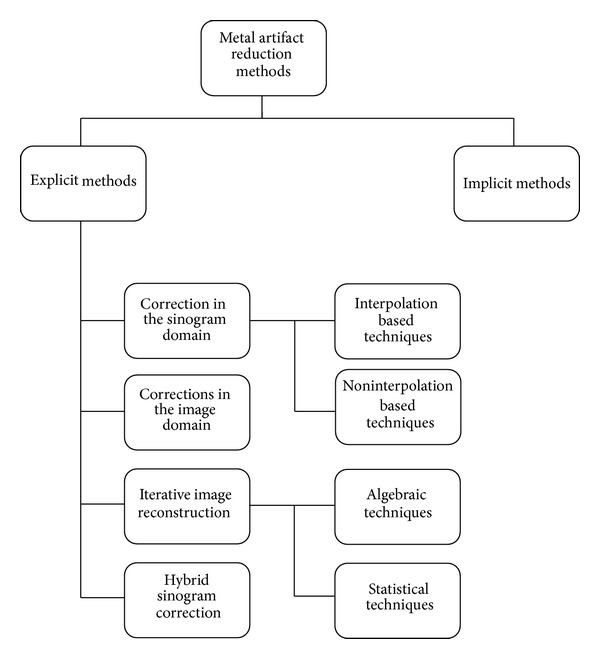
Classification of metal artifact methods.

According to the authors, explicit techniques can be grouped into four categories (Figure 1): corrections in the sinogram domain, corrections in the image domain, iterative reconstruction algorithms, and hybrid sinogram correction. Corrections in the sinogram domain can be further divided into interpolation-based and noninterpolation-based sinogram correction techniques.

Some of the interpolation-based algorithms are described in [10, 12, 13]. The basic idea consists in finding projection bins which are influenced by the metal in the raw data and then replacing them with values calculated using the linear interpolation method. The affected projection bins are usually found with simple thresholding techniques. After the correction is performed in the sinogram domain, data is back-projected in order to reconstruct the image.

Noninterpolation-based algorithms use different approaches instead of interpolation, such as Monte Carlo simulation [14].

An approach which uses bilateral filtering is presented by Cheng and Liu [15]. First they start with the metal region identification. This was achieved by thresholding, since pixels in the metallic region have higher pixel values. In addition to thresholding, a connectivity test was conducted to obtain the regions with metal. Once identified, metal traces were reprojected to the projection domain and the pixel values in those regions temporarily set to pixel values of neighboring soft tissues. Filtered image was then forward projected and new values were assigned to affected projection bins. Finally, segmented metallic objects were superimposed back onto the reconstructed image. Methods in the sinogram domain generally give better results than other methods. On the other hand, working with raw data is often cumbersome due to its large size and the need to account for proprietary data formats and specific system geometries.

Corrections in the image domain deal with reconstructed images. Kennedy et al. [16] proposed a method which uses a Bayes classifier applied in an annular region around groups of saturated pixels. Different threshold values were used for detection of pixels representing flare, photon deficiency, and metal. Detected pixel values were substituted with pixel values of soft tissue or bone. Sohmura et al. proposed an approach [17] in which a dental cast model is used to replace regions affected with artifacts. The drawback of image-domain methods is that affected pixel values are most often replaced with constant values leading to further degradation of the visual image quality. However, working with reconstructed images is faster and more generic, as it can be applied across different acquisition systems.

Iterative reconstruction is carried out after the detection of affected projection bins. In CT, it is not used as widely as in PET. The main reason for this is that data-sets in CT are much larger making the process computationally very intensive. Iterative reconstruction algorithms include algebraic and statistical techniques. Several methods have been proposed in [18–20].

In addition to the techniques mentioned, several hybrid techniques exist, which try to combine different basic methods. Nuyts and Stroobants proposed a method which is a combination of linear interpolation and iterative reconstruction [21]. Another hybrid approach is presented by Tuy, where the author used a combination of interpolation and noninterpolation-based sinogram correction [22].

All of the methods discussed have the same limitation, as they all use only data available from CT. Since metal objects create regions with missing projection data, their results strongly depend on the size and number of metal implants present in the patient's body. Recent trend in availability of trimodality systems provides an opportunity to use complementary MR data for reduction of artifacts in CT images. This area is not yet sufficiently explored and one of the first efforts in this field is presented by Delso et al. [23]. 

In this paper, we propose a new method for the reduction of metal artifacts in CT images using additional information obtained from MR data. This is a fully automatic method which detects metal implants and reduces streaking artifacts. 

## 2. Material and Methods

Our algorithm is tested on data from two patients who were referred for a PET/CT scan and volunteered for additional MR scan. Before PET scan, the patients had a resting period after the injection of ^18^F-FDG. During that period an MR scan was performed. All clinical procedures were approved by the local ethics committee.

The trimodality system consists of GE Healthcare Discovery 690 PET/CT and Discovery 750 w MR scanner. The MR is located in a room next to PET/CT scanner. The MR table is undockable and it was moved with a shuttle device (Innovation Design Center, Thalwil, Switzerland) to the PET/CT room. With this possibility, the patient did not have to alter his position.

The PET/CT acquisition followed the standard clinical protocol for an oncological whole-body study (helical CT scan, 120 kV, 20,100 mA with auto-mA intensity modulation, convolution kernel “GE-Standard” (low-pass), 512 × 512 matrix, 0.98 × 0.98 × 3.27 mm^3^, 2 min PET stations, ~300 MBq FDG).

The MR sequence was a LAVA-Flex, a 3D fast spoiled gradient echo sequence. It is a two-point Dixon method that provides four separate contrasts (water, fat, in-phase, and out-of-phase) in one single acquisition. This sequence is routinely used on all whole-body trimodality patients to provide T1-weighted whole-body images for anatomical localization.

The Integrated Registration tool which is available at the GE Advantage Workstation was used to verify the proper alignment of the different datasets.

Our method can be separated in two parts: detection of metal artifacts and correction algorithm.

### 2.1. Metal Artifact Detection

The detection of artifacts in the image is achieved using Otsu's thresholding method [24]. This method belongs to the class of clustering-based thresholding methods, which means that the gray level data undergoes a clustering analysis, with the number of clusters always set to two. The approach is based on the concept of finding the threshold that minimizes the weighted within-class variance and operates directly on the gray level histogram. The method produces good results when the number of pixels in each class is balanced.

To obtain the threshold, for each potential threshold consider the following.

- According to the threshold, the pixels are separated into two clusters.
- The mean of each cluster is found.
- The difference between the means is squared.
- The number of pixels in one cluster is multiplied by the number in the other.

**Figure 2:**
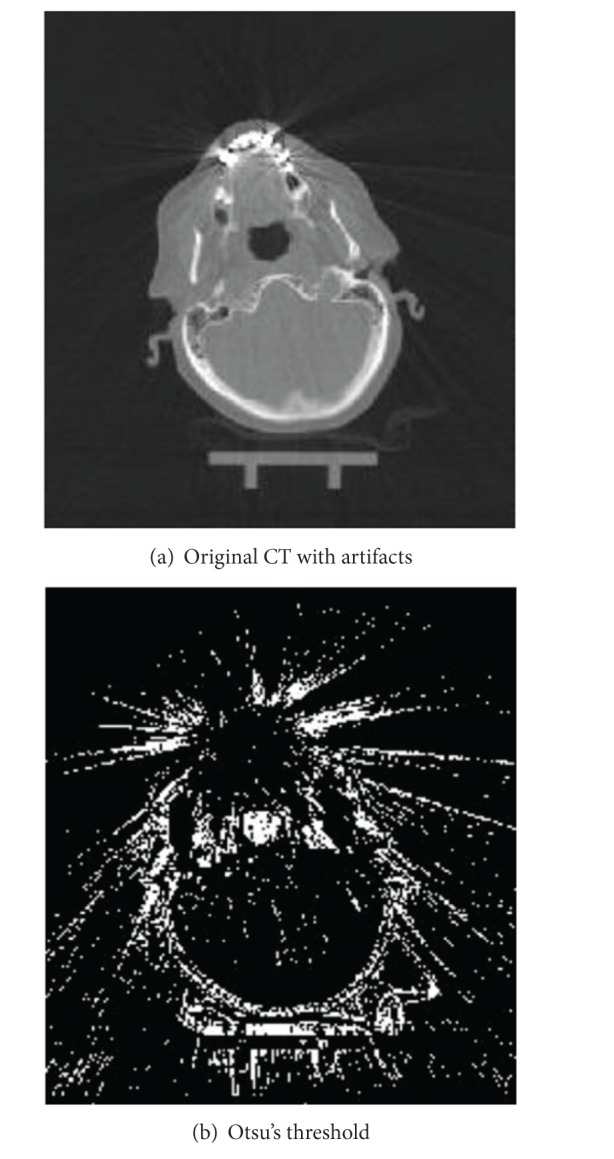
Original CT image with dental streaking artifacts (a) and detected artifacts with Otsu's thresholding method (b).

The optimal threshold is the one that minimizes the within-class variance, or, conversely, maximizes the between-class variance. As the number of classes increases, selected thresholds become less valid. Experimenting with images with artifacts, we found that 50 classes are an optimal choice for metal region identification.  Figure 2(b) shows the result after setting the number of classes to *n* = 50 and isolating classes that do not represent artifacts. As it can be seen in Figure 2, the original CT image contains severe dental artifacts with streaking lines (Figure 2(a)). After Otsu's thresholding, the obtained mask shows that the streaking lines are detected (Figure 2(b)).

### 2.2. Correction Algorithm

Once the artifact regions are identified, we use the correction algorithm to modify pixel values and thus reduce the effects of present metal objects. The algorithm proposed in this paper changes the values of corrupted pixels, while uncorrupted pixels are left undisturbed. Corrupted pixels are indicated by white pixels in the threshold mask.

CT images have 512 × 512 gray scale pixel resolution.

The proposed algorithm is as follows.

*Step  1.* A two dimensional 5 × 5 window is slid over the CT image. 

*Step  2.* If the central pixel is a corrupted pixel, then its value is changed according to the next step. Otherwise, it is considered uncorrupted and the window is slid to the next position. 

*Step  3.* The algorithm finds the same pixel position on the corresponding MR image and then looks for the position of the pixel most similar to the central pixel in the 5 × 5 window. 

*Step  4.* We examine the CT image pixel with the same position as the newly found MR pixel position. If that pixel is uncorrupted in CT, then its value is assigned to the central corrupted pixel in CT. In case that pixel is also corrupted in CT, we look at the next most similar pixel in MR image and repeat this step until we find an uncorrupted pixel. 

**Figure 3:**
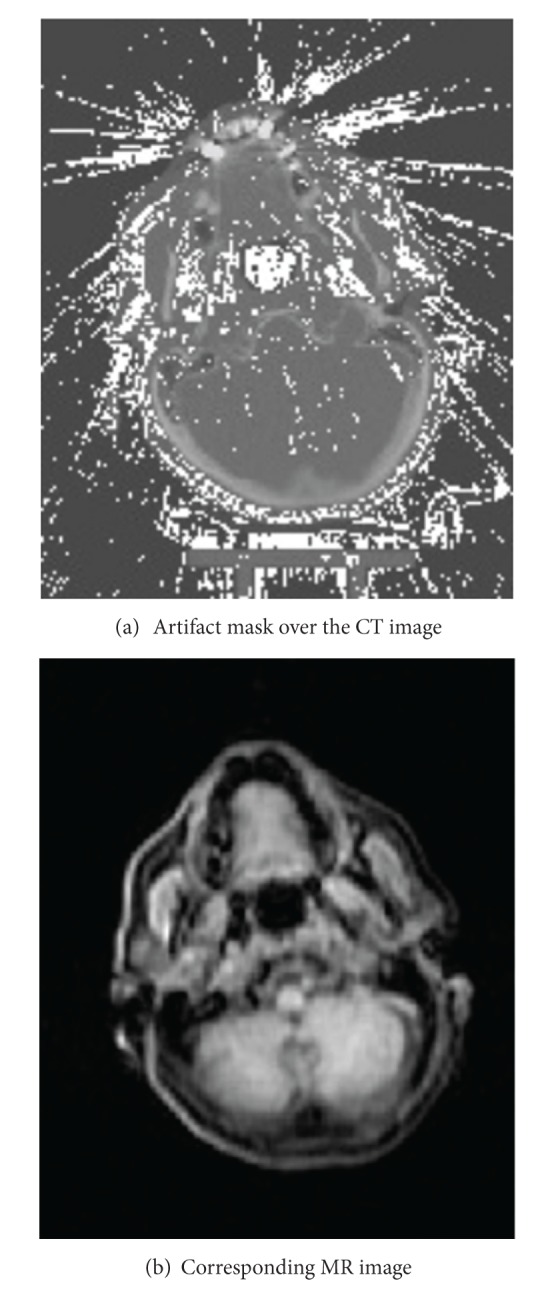
Detected artifacts on CT image (a) and corresponding MR image (b).

Figure 3 shows the artifact mask over the CT image with artifacts (Figure 3(a)) and the corresponding MR image (Figure 3(b)), respectively. These two images were used as an input to the proposed algorithm which has been implemented in Matlab.

Since the algorithm cannot be applied to border pixels, the first two and the last two columns are replicated at the front and rear end of the CT image, respectively. Similarly, the first two and the last two rows are replicated at the top and bottom of the CT image, respectively.

### 2.3. Validation of Results

In order to test the possibilities of the proposed method, we performed a simulation study in which we compared our results to results obtained through linear interpolation. Linear interpolation represents a standard method for missing projections reconstruction. The simulation study was carried out using a cone-beam projection/reprojection tool implemented in Matlab. CT data without dental implants was used to achieve this. Different numbers and sizes of implants were introduced and the images in dataset were forward projected to simulate the effects of dental implants. Resulting images were then back-projected and corrected using linear interpolation method and the proposed method.

## 3. Results and Discussion

The presented approach is an image-based technique for metal-artifact reduction, which differs from other algorithms mainly in the approach to the correction of metal artifacts. Typically, the missing projection data is artificially generated, while our method uses information from the complementary MR image to generate the missing data. However, MR images may suffer from signal voids which are caused by the implants. The MR artifacts are present around the implant and they can vary depending on both size and composition. This lack of information represents the limitation of the current version of the method and it could be overcome in the future using alternative MR sequences which are capable of imaging near metal. Carl et al. [25] investigated the potential of combining ultrashort echo time with multiple acquisition with variable resonance image combination to image tissues adjacent to metallic implants and they found out that it is possible to significantly reduce typical artifacts near metal.

**Figure 4:**
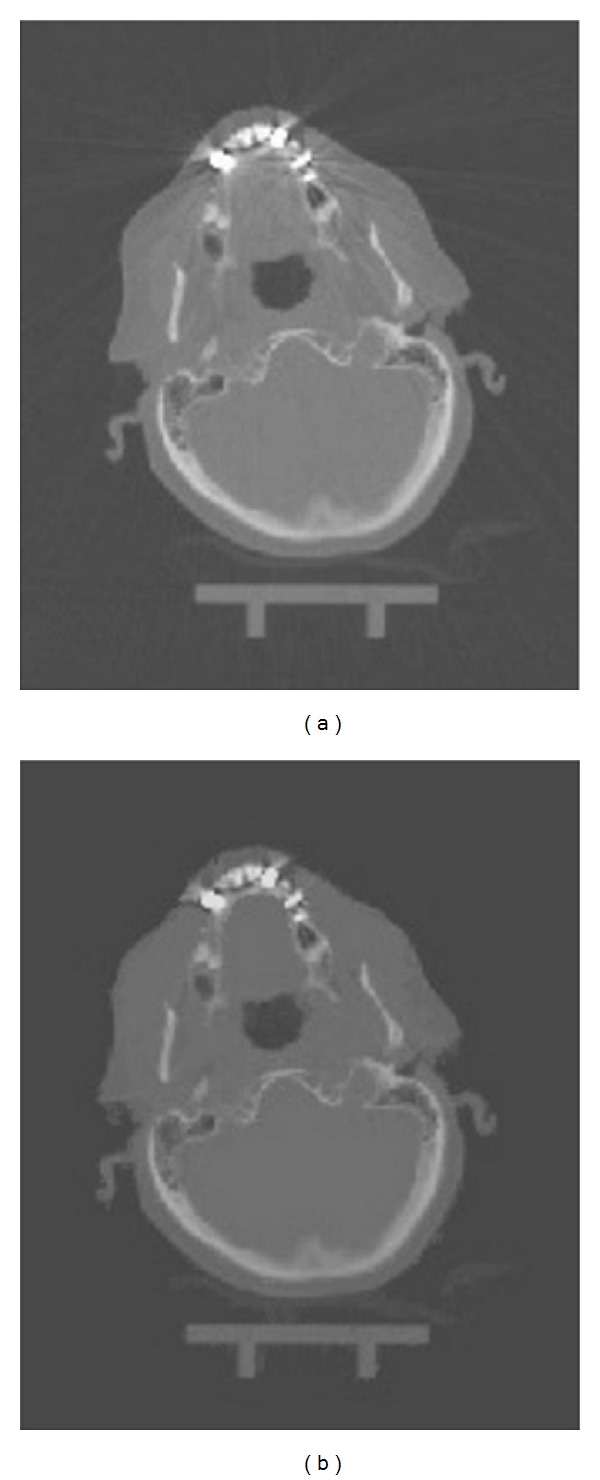
Axial view of CT dataset before (a) and after correction (b).

Figure 4 shows a CT image before and after correction based on our method. The resulting images do not have secondary artifacts, which are very often an issue with other image based artifact reduction methods. It can be observed that our method is particularly effective in reducing streaking lines in the area outside the skull and, therefore, also suitable for the creation of PET attenuation maps.

CT images are commonly used for attenuation maps for correcting PET data and therefore it is important to provide CT images with artifacts reduced as much as possible. MR-based attenuation correction is not as straightforward as CT attenuation correction which allows the estimation of 511 keV attenuation maps from CT images. However, there are some studies which try to utilize MR-based attenuation correction [26, 27]. The most difficult task in creating attenuation maps from MR images is the discrimination of bone tissue. This is an objective of current research.

**Figure 5:**
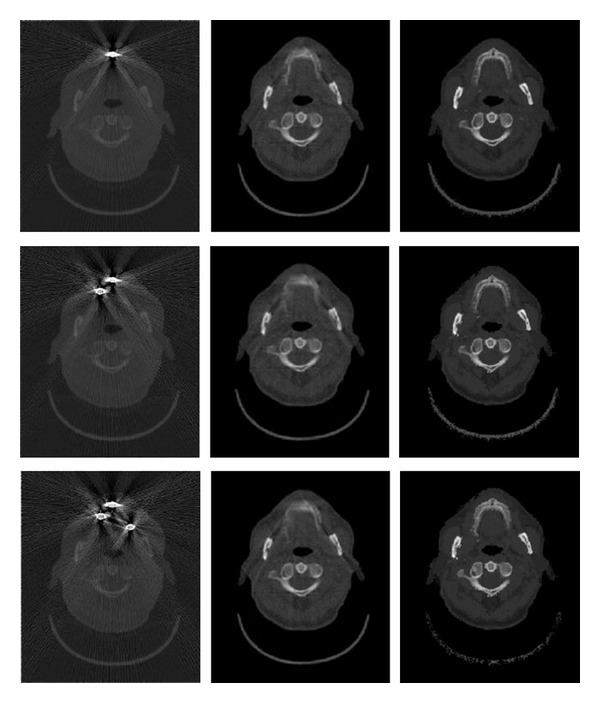
Axial view of CT dataset with simulated implants is in the first column, the second column shows the results obtained with linear interpolation method, and the results of the proposed method are presented in the last column.

The results from the simulation study are shown in Figure 5. Images in the first column contain simulated artifacts, the second column contains images reconstructed with the linear interpolation method, and the third column shows images which are the result of the proposed method. One should note that the performance of the linear interpolation method decreases as the number of implants increases.

A study conducted on 152 patients [23] showed that most of patients (80 percent) had dental fillings. That is the reason why we primarily focused on dental implants. Nevertheless our approach could also be applied in cases other than dental fillings, such as subclavian ports or hip implants.

In certain cases, we had incorrect registration between CT and MR images, which had, as a consequence, undesired occlusion of the airways. Image misregistration can occur due to hardware and patient-induced errors [28]. Hardware errors are related to mechanical tolerances of the shuttle system. Rigid transformations can describe and make a good approximation of hardware registration errors. On the other hand, patient-induced errors have nonrigid nature and they depend on patient's condition, his comfort, and anxiety. It can be noticed that these factors have more significant impact as the time increases between the individual scans. A possible solution for this is to use on both scanners accurate and reproducible laser landmarking of the patient. This method can almost eliminate registration errors along the axial direction. Accurate table height adjustment can minimize the misregistration along lateral direction. Samarin et al. [29] reported recently that the mean offset between MR and PET/CT was below 1 centimeter. The Integrated Registration tool was used to further refine image registration. The image registration is done by registering the MR data towards CT, assuming accurate geometric calibration of the PET/CT system.

However, this method has some weaknesses. The achieved improvement is limited by bone tissue extraction. It is a common problem to isolate the bone tissue from thresholding. Since bone structures have high density, they appear bright on CT images and it is hard to separate them from metal implants which often occur in their vicinity. MR images could help to overcome this problem. One possible solution is to use fast MR sequences which are capable of detecting bone tissues and in that case MR images could be used to isolate high-density structures other than metal.

In order to test the robustness and practical applicability of the proposed method, a larger population of patients is required. We could also examine how the position, size, and number of metal objects influence the results.

## 4. Conclusion

In this paper, we proposed a new method for metal artifact reduction. A combination of Otsu's thresholding and paired MR images is used to achieve the correction of CT images.

The method was shown to be effective in eliminating streaking artifacts that originate from metal. Our approach is fast and simple and it is fully automated. There is no need for manual interaction, nor for defining the region of interest.

Another benefit of the proposed method is that there is no need to work on complex raw CT data. Currently, the application of the proposed methodology is limited to a relatively small number of clinical centers which have trimodality systems. However, the number of such institutions should increase significantly in the near future.

Further research will be focused on soft tissue reconstruction and on a larger population of patients. Also, working in the sinogram domain instead of image domain should improve our results and help to introduce this method in everyday clinical practice.
